# Academic Career Design: A Model for Personalizing Today's Academic Neurology and Neuroscience Work Environment

**DOI:** 10.1212/NE9.0000000000200266

**Published:** 2025-10-29

**Authors:** Rachel Marie E. Salas*, Cherie L. Marvel*, Thabele M. Leslie-Mazwi, Jennifer Bickel, Charlene E. Gamaldo

**Affiliations:** 1 Johns Hopkins University, Baltimore, MD;; 2 University of Washington, Seattle; and; 3 Moffitt Cancer Center, University of South Florida, Tampa.

Since the 2020 pandemic, the journey of academic faculty has naturally evolved within the virtual space. This Viewpoint explores some of the new variables, factors, considerations, and potential implications of this new reality for academic medicine, particularly in our field of neurology.

## Academic Career Design: Customized Life Design Approach

Whether one is engaged in teaching, research, or clinical practice, the digital realm provides a customizable platform to offer unique avenues for enhancing collaboration and communication (Figure, A). This tailored approach ensures that each academic professional can contribute effectively to their field while enjoying the flexibility and personalization that remote or virtual work environments offer.

**Figure:**
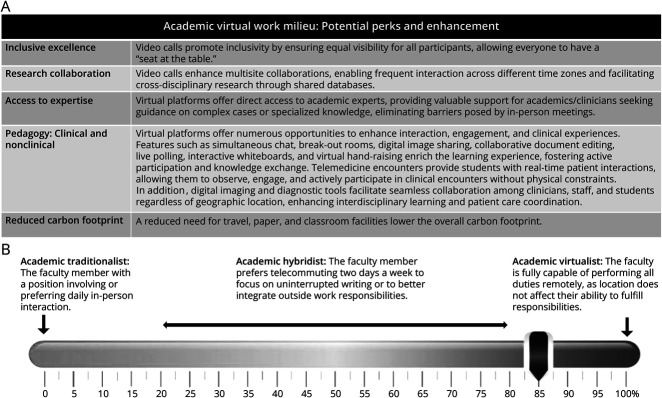
Academic Virtual Work Milieu: Potential Perks and Enhancements.

The Academic Career Design (ACD) model draws inspiration from the principles of life design.^1^ The life design paradigm enables individuals to intentionally and creatively shape their lives by identifying goals, exploring options, and experimenting with various paths. Life design principles can provide a framework for academic faculty to identify their career goals, define success, and develop the best strategies to achieve it. To realize a practical ACD approach that aligns with the goals; mission; and success metrics for faculty, staff, learners, patients, and members of the academic community, we have identified the following variables that need to be considered at a minimum: (1) workplace optimization and feasibility, (2) patient care needs alignment, (3) leadership advocacy, (4) staff and team operational support (Table).

**Table:** Considerations for Academic Work

Essential element	Domain	Successful examples	Considerations
Feasibility	Telemedicine	Stroke, epilepsy, sleep, headache, and movement disorders	Not applicable to all specialties (e.g., Interventional Neurology), regulatory requirements, and compliance issues related to telemedicine
	Teaching	Didactics and interviews (e.g., residency)	Could slow the formation of meaningful academic relationships, some teaching is best in person (e.g., examination skills)
	Research	Collaboration with other institutions, online data collection, and increased mentor/mentee opportunities with learners off-campus	Reduces the value of impromptu “hallway” conversations
	Patient value	Telemedicine visits are comparable to in-person visits for history assessment and patient connection	Less effective for neurologic examinations
Patient care alignment	Patient safety	Telemedicine has increased benefits in infection control, accessibility, continuity of care with more regular follow-up visits, reduced travel burden, quicker access to care, enhanced multidisciplinary care, improved family engagement and presence at visit, and convenience, particularly for patients with pain, reduced mobility, or declining cognitive issues	Technical aspects and access challenges
Leadership advocacy	Leadership	Supporting a flexible work model with mutual benefit to faculty members and the department; approval facilitates the various practical considerations for virtual work (licensing, equipment, regulatory needs, scheduling, administrative support, etc.)	In-person FaceTime may better suit the needs of the institution for certain functions, meaning that 100% virtual is not feasible or supported by leadership
Staff/team operational support	Acceptance	Fosters a collaborative environment and demonstrates understanding and respect of colleagues' work-life interactions and arrangements; team alignment can positively influence faculty satisfaction and retention	Building of community has to be intentional; there may be challenges for all members of the team to remain connected, engaged, and integrated into the academic community, regardless of their work environment arrangement

## Challenges and Considerations

Academia recognizes the importance of fostering a harmonious work-life integration philosophy to retain and promote faculty talent. At the same time, academic institutions must balance this individualized approach with meeting the diverse needs and preferences of their entire faculty, staff, learners, and institutional goals. Balancing the individual needs of students with those of the academic community as a whole can be a source of tension and conflicting interests. As a result, the final product in an individualized ACD must strike a balance between preference and practicality. Leadership can help identify guidelines for duties that require a physical presence. Perceived or actual inequities may arise if some roles are more conducive to virtual work than others, potentially leading to a perceived inequity. Leadership's transparent criteria and virtual work policies, in combination with flexibility and openness to pilot new processes, are necessary, allowing the opportunity to consider the appropriate degree of leadership advocacy. Leadership could provide additional support for jobs requiring a greater in-person presence (e.g., a commuter stipend) to ensure greater equity. Methods such as culture surveys; department meetings; and focus groups are paramount in helping to keep the finger on the pulse of faculty, team, and department satisfaction and engagement.

Flexibility and autonomy in hybrid models can only thrive when paired with accountability, yet too often, broad rules are implemented rather than having direct conversations with underperforming individuals. A shift in focus toward results over hours-in-office could encourage a culture that supports flexibility while maintaining accountability. Some neurologists and academics may experience a disconnect with the shift to virtual and hybrid office models. Having a designated office space holds symbolic value, representing status; recognition; and a sense of belonging within an institution, center, or group. Losing this dedicated space can make some individuals feel devalued and, in some cases, even disposable. Drawing inspiration from the “WeWork” model, some institutions have developed shared community, team, and individual touchdown spaces that can be reserved on a recurring schedule.^2^ This approach enables individuals to establish a mutually agreed-upon frequency for in-person FaceTime meetings that strikes a balance between connectivity and convenience factors. Moreover, this approach also helps with the longstanding “space crunch” that most universities face. From an educational standpoint, educators can bridge the virtual gap in teaching neurology by combining high-quality video demonstrations, interactive simulations, structured peer feedback, and hybrid models that reinforce hands-on skills through targeted in-person experiences.

For leadership, specific challenges also must be considered. Onboarding new remote employees must be specifically designed to achieve the goals of community building and orientation. Security risks, such as data breaches and cyberattacks, can escalate without additional measures in place. Insurance coverage, funding models for billing practices, and salary coverage are other considerations. Academic leadership would also need to consider best practices to ensure adequate, transparent, and effective monitoring of care and research quality. Finally, there may be an increased administrative burden to oversee state licensure, clinical billing, and purchasing supplies and materials. While all these considerations are essential, it is important to note that many positive benefits at the individual level are also advantageous. Crucially, more flexibility for academic clinicians often translates into more flexibility for patients as well, with enhanced hours and access for patients, learners, collaborators, and the public to connect with an academic institution and its members. Finally, virtual networks and communities of practice have thrived across many fields by fostering connection, mentorship, and collaborative learning beyond geographic constraints. Their successful transition to online platforms highlights the potential for similar strategies in clinical neurology, neurology education, especially for professional development and peer learning.

## Conclusion

The academic workplace environment has and will continue to evolve, with the preferences of providers, patients, learners, and administrators enabled by the integration of technology. The era of the “one size fits all” academic neurology work model is in the past and now calls for customized approaches that best meet the mutual needs of all members and stakeholders of our academic neurology community. Using principles of an ACD paradigm of personalization, flexibility, and goal alignment can provide a guide for striking the most harmonious balance. Dynamic and ongoing efforts will ensure that academia continues to nurture and develop today's and tomorrow's talent.
